# A study protocol for implementing Canadian Practice Guidelines for Treating Children and Adolescents with Eating Disorders

**DOI:** 10.1186/s43058-023-00538-9

**Published:** 2024-01-05

**Authors:** Jennifer L. Couturier, Melissa Kimber, Catherine Ford, Jennifer S. Coelho, Gina Dimitropoulos, Ayisha Kurji, Jonathan Boman, Leanna Isserlin, Jason Bond, Chelsea Soroka, Anna Dominic, Ahmed Boachie, Gail McVey, Mark Norris, Nicole Obeid, David Pilon, Wendy Spettigue, Sheri Findlay, Josie Geller, Seena Grewal, Joanne Gusella, Monique Jericho, Natasha Johnson, Debra Katzman, Natalie Chan, Chloe Grande, Maria Nicula, Drew Clause-Walford, Anick Leclerc, Rachel Loewen, Techiya Loewen, Cathleen Steinegger, Elizabeth Waite, Cheryl Webb, Melissa Brouwers

**Affiliations:** 1 McMaster Children’s Hospital, McMaster University, 1200 Main St W, Hamilton, ON L8N 3Z5 Canada; 2 Eating Disorders Ontario, Toronto, Canada; 3 University of British Columbia, Vancouver, Canada; 4 University of Calgary, Calgary, Canada; 5 University of Saskatchewan, Saskatoon, Canada; 6 University of Manitoba, Winnipeg, Canada; 7 University of Ottawa, Ottawa, Canada; 8 McGill University, Montreal, Canada; 9 Dalhousie University, Halifax, Canada; 10 Memorial University, St. John’s, Canada; 11 University of Toronto, Toronto, Canada; 12 Private Practice, Halifax, Canada; 13 Lived Experience Advisor, Toronto, Canada; 14 McMaster Children’s Hospital, Hamilton, Canada

**Keywords:** Eating disorders, Adolescence, Guidelines, Implementation strategy, Clinical practice

## Abstract

**Background:**

Eating disorders have one of the highest mortality rates among psychiatric illnesses. Timely intervention is crucial for effective treatment, as eating disorders tend to be chronic and difficult to manage if left untreated. Clinical practice guidelines play a vital role in improving healthcare delivery, aiming to minimize variations in care and bridge the gap between research and practice. However, research indicates an active guideline implementation approach is crucial to effective uptake.

**Methods:**

Mixed methods will be used to inform and evaluate our guideline implementation approach. Semi-structured focus groups will be conducted in each of the eight provinces in Canada. Each focus group will comprise 8–10 key stakeholders, including clinicians, program administrators, and individuals with lived experience or caregivers. Qualitative data will be analyzed using conventional content analysis and the constant comparison technique and the results will be used to inform our implementation strategy. The study will then evaluate the effectiveness of our implementation approach through pre- and post-surveys, comparing changes in awareness, use, and impact of the guidelines in various stakeholder groups.

**Discussion:**

Through a multifaceted implementation strategy, involving the co-creation of educational materials, tailored training, and context-specific strategies, this study intends to enhance guideline uptake and promote adherence to evidence-based practices. Our study will also contribute valuable information on the impact of our implementation strategies.

**Supplementary Information:**

The online version contains supplementary material available at 10.1186/s43058-023-00538-9.

## Lay Abstract

This protocol outlines a comprehensive research study aimed at evaluating an implementation approach for the uptake of the Canadian Practice Guidelines for Treating Children and Adolescents with Eating Disorders across Canada. Eating disorders (EDs) have a significant impact on mental and physical health, necessitating evidence-based and timely interventions. To address gaps in service, we propose to use focus groups to identify barriers, facilitators, and implementation strategies for guideline uptake. We will engage stakeholders, including clinicians, administrators, and individuals with lived experience, to co-create tailored educational materials which will then be disseminated. The impact of the implementation approach will be evaluated through pre- and post-implementation surveys. This study aims to enhance the availability and utilization of evidence-based treatments for children and adolescents with EDs, ensuring equitable and improved care nationwide.

**Supplementary Information:**

The online version contains supplementary material available at 10.1186/s43058-023-00538-9.

Contributions to the literature

- Our findings will contribute to knowledge regarding implementation strategies for clinical practice guidelines.
- Participants in focus groups will generate guideline implementation strategies for diverse populations.
- Survey data will indicate which guideline implementation strategies were most effective.

## Background

Eating disorders (EDs) cause significant impairment in mental and physical health [1] and have one of the highest mortality rates of all psychiatric illnesses [2]. These disorders are known for being chronic and difficult to treat, especially if intervention is not received within the first 3 years of symptom onset [3]. EDs are common, with Canadian community surveys reporting 2.2% of males and 4.5% of females under 18 years of age meeting criteria [4]. The COVID-19 pandemic has resulted in long waiting lists and fragmented care for children and youth with EDs, amplifying problems in the system that existed prior to the pandemic [5].

Clinical practice guidelines are “systematically developed statements to assist practitioners in making decisions about appropriate health care for specific clinical circumstances” [6]. Guidelines are intended to reduce variability in care and to decrease the gap between research and practice [7]. Adherence to treatment guidelines has the potential to mitigate system inefficiencies. It is estimated that 30–40% of patients receive treatment that is not evidence-based, and 20–25% receive treatment that is not needed or potentially harmful [8]. Guidelines can help to reduce these statistics, but must be implemented successfully in order to result in practice change.

Published just prior to the onset of the pandemic, our team developed Canadian Practice Guidelines which outlined evidence-based treatments for children and youth with EDs [9]. Our panel of stakeholders came together again during the pandemic to publish a virtual care addendum to these guidelines [10]. Although the guidelines are published in an open-access journal and efforts have been made to distribute our findings, a systematic approach to implement our guidelines has not been developed nor studied. This project aims to pull together key stakeholders to jointly develop an implementation strategy and to evaluate the uptake of these guidelines on a wider scale across Canada.

### Barriers to guideline implementation

A recent scoping review on barriers to guideline implementation divided themes into three categories: (1) personal factors, (2) guideline-related factors, and (3) external factors [11]. Personal factors included personal views and beliefs about the guideline recommendations. Guideline-related factors included lack of clarity of the guideline, lack of conciseness, or ambiguity of the guideline [12–14]. External factors included the complexities of the treatment(s) proposed and whether clinicians needed training, as well as the ability of the organization to adopt the interventions [15–17]. Successful guideline implementation requires an examination of these factors as a first step.

### Strategies to enhance implementation

A recent systematic review of strategies for the implementation of clinical practice guidelines found that the most frequently studied interventions were educational materials, educational meetings, reminders, academic detailing, and audit and feedback, with many studies using a multi-faceted approach [18]. A separate scoping review found that dissemination, education and training, social interaction, decision support systems, and standing orders were central elements of successful strategies [11]. These authors suggest that implementation strategies be tailored to the context and target audience [11]. In addition, the strategy should be multifaceted and should address practitioners’ knowledge and attitudes in order to be effective [11]. Stakeholders must be involved in discussing barriers and developing strategies, and a patient version of a guideline may help to support the implementation process [11]. Furthermore, the adherence of professionals and organizations to guidelines can be improved when they are developed locally or adapted [19]. 

Our guideline research to date has focused on the synthesis of knowledge and production of Canadian Practice Guidelines [9] and a virtual care addendum [10] for treating children and adolescents with EDs. Studying the implementation of these guidelines is the next logical step in our knowledge to action plan. This includes examining barriers and facilitating factors to guideline implementation, developing a guideline implementation strategy, and completing a preliminary evaluation of this strategy. Thus, our research questions would be as follows: (1) What are the barriers and facilitators to the implementation of the Canadian Practice Guidelines (and Virtual Care Addendum) for Treating Children and Adolescents with Eating Disorders? (2) What strategies could be used to enhance the implementation of these treatment guidelines? (3) What additional strategies could be used to enhance implementation for the most vulnerable and diverse populations of children and adolescents with eating disorders? (4) Was our implementation approach effective?

## Methods/design

Our work will combine principles of implementation science [20] with a learning health systems approach [21] in order to involve various stakeholders within several learning communities with a common goal to improve health outcomes for our target population of youth with eating disorders. We will execute a concurrent mixed-method research design. Qualitative and quantitative strands of data will be collected in parallel for the purpose of gaining a more robust understanding of guideline implementation barriers and facilitators, as well as strategies to enhance uptake. The qualitative strand will follow a qualitative descriptive design [22]. We will conduct focus groups as well as an online survey in order to answer our research questions. Our methodology and reporting will be guided by two checklists. We will use the SQUIRE (Standards for QUality Improvement Reporting Excellence) checklist when writing our report [23]. The TIDieR (Template for Intervention Description and Replication) Checklist will be used to facilitate a comprehensive assessment of our intervention’s efficacy and implementation strategies [24].

In terms of the qualitative component, eight focus groups will be conducted across Canada (British Columbia, Alberta, Saskatchewan, Manitoba, Ontario, Quebec, Nova Scotia, and Newfoundland). Unfortunately, ED programs do not currently exist in Nunavut, New Brunswick, Prince Edward Island, Northwest Territories, and the Yukon [5]. However, the focus groups will address questions of how to reach remote areas, including those without dedicated ED programs. The interview guide will also address questions related to the most vulnerable youth—those from Indigenous communities, Black and racialized groups, and gender-diverse populations (see Additional file 1 for the semi-structured focus group interview guide). Focus group participants in each of the eight provinces will also discuss guideline implementation barriers and facilitators, as well as strategies for uptake. Focus groups will involve 8–10 key stakeholders which will include 3–4 clinicians, 2–3 program administrators, and 3–4 persons with lived experience. Focus groups will be video-recorded using Zoom for Healthcare, transcribed verbatim, and analyzed using NVivo software.

Concurrently, we will use survey methodology (Dillman Tailored Design Method) [25] to create and conduct an online survey at two timepoints: baseline and then at the post-implementation period. This will allow us to evaluate our guideline implementation strategy. The baseline survey will include some additional elements on barriers and facilitators, whereas the follow-up survey will include views on our implementation strategy (see Additional file 2 for the pre- and post-survey). The survey will target a group of stakeholders belonging to key membership groups [Eating Disorders Association of Canada (EDAC) and relevant sections from the Canadian Pediatric Society (CPS: Mental Health, Adolescent Health and Community sections)] as well as those with lived experience through the National Initiative for Eating Disorders (NIED), the National Eating Disorders Information Centre (NEDIC), and Body Brave. In terms of EDAC and CPS, the membership will be emailed internally and provided with a link to the survey. In terms of NIED, NEDIC, and Body Brave, the survey will be advertised on their websites and social media with a link. The survey will collect identifying information and assign a unique identifier so that we can link pre- and post-data. The post-survey will only be sent to those who completed the pre-survey.

It is anticipated that through the focus groups and surveys, stakeholders will identify several barriers and facilitators, as well as strategies to implement our guidelines. The barriers might include personal factors (attitude toward specific recommended treatments which may include some ambivalent views based on our prior research [26]), guideline factors (length of our guideline is 80 pages for the Canadian Practice Guidelines and 40 pages for the addendum, both of which could be shortened), and external factors (waiting lists are very long [5]). In order to mitigate the guideline factors, we anticipate creating educational materials as part of our guideline implementation strategy including a guideline synopsis and patient guide. We also anticipate designing tailored and context-specific educational strategies to reach clinicians, administrators, policymakers, and advocacy groups, such as educational videos. Once created, these materials will be emailed to everyone who participated in the focus groups and survey, and links will also be posted on various websites (NIED, NEDIC, Body Brave, EDAC, CPS sections).

### Barriers and facilitators

The focus groups will discuss the barriers and facilitators (personal, guideline, and external) [11] to our guideline implementation, as well as implementation strategies, focusing on the elements of the Lavis knowledge transfer framework: (1) the message, (2) the messenger, (3) the target audience, (4) strategies and infrastructure, and (5) evaluation [27], and ideas for reaching the most vulnerable populations. Focus groups will be evaluated qualitatively using the method of fundamental qualitative description [22].

In addition, an online survey using Qualtrics will use a Likert scale to evaluate the importance of certain anticipated barriers and facilitators (strongly disagree, disagree, neutral, agree, strongly agree), as well as, open-ended questions to elicit further information on these topics. The survey will also be evaluated qualitatively by examining the text that participants write within the survey. The text will be analyzed in the same fashion as the transcripts from the focus groups.

### Implementation effectiveness

Pre- and post-surveys will be sent to participants mentioned above in order to examine change and capture any improvement in awareness, use, and impact of the guidelines prior to and following our anticipated implementation strategy. The items on awareness, use, and impact will be rated on a 5-point Likert scale (none, little, moderate, high, and very high). Upon enrollment, each participant will be assigned a unique identifier and will be tracked and matched to evaluate change in scores for awareness, use, and impact. Post surveys will also contain open-ended questions asking for feedback on the implementation approach and the text will be coded in the same qualitative fashion used for the focus groups.

### Analysis

#### Qualitative

Transcripts from the eight focus groups will be analyzed using conventional content analysis and the constant comparison technique [28]. Specifically, iterative reviews of text within and across transcripts will be completed by two members of the research team in order to identify the salient content characterizing the barriers and facilitators to guideline implementation, as well as strategies for implementation. Open-ended questions on the online survey asking about these same topics will also be analyzed qualitatively using the same method.

#### Quantitative

Pre- and post-online surveys will be compared using paired *t*-tests on items rating awareness, use, and impact of our guidelines.

#### Sample size considerations

For our qualitative data, a sample of eight focus groups with ten participants each is standard for other qualitative studies in the field [29]. Our total potential sample for the online survey will consist of 845 members of the various sections of CPS plus EDAC, plus an unknown number we will recruit through websites associated with NIED, NEDIC, and Body Brave. Even with an expected recruitment rate of 50% through EDAC and CPS, we will still have a large enough sample to answer our quantitative research questions on awareness, use, and impact of our guidelines. Using a sample size calculation from “sample-size.net” and a paired *t*-test analysis, assuming an effect size of 0.5, standard deviation of the change in score of 2 on the 5-point Likert scale, alpha of 0.05, and beta of 0.2, a sample of 128 would be required.

### Study status

At the time of this report, our team has received ethics approval and is beginning to form focus groups for the qualitative component of the study. We are also preparing to send out the pre-implementation survey. Recruitment has not yet started.

## Discussion

Research on guideline implementation is needed in general, but also more specifically in the field of eating disorders. The gap between research and practice can be narrowed by clinical practice guidelines; however, they must be actively implemented in order to result in change in knowledge and practice. This study leverages a network of clinicians, methodologists, and knowledge users dedicated to improving the health care system for children and adolescents with EDs. This study will directly benefit stakeholders across Canada by providing educational tools on treatments for children and adolescents with EDs. This will ensure that knowledge on evidence-based treatment for children and adolescents with EDs is widely available to all Canadians.

This study will also provide valuable information on guideline implementation strategies. Little is known about how best to reach clinicians in the field of eating disorders. Particularly following the COVID-19 pandemic with dramatic increases in eating disorder cases and staff turnover, these are challenging times for implementation. However, there is an opportunity to reach new staff and to capitalize on programs that are expanding under the influx of new cases.

A recent systematic review summarizing the state of the literature on guideline implementation has indicated that pre-planning with stakeholder engagement and identification of barriers is a common strategy employed in guideline implementation studies [30]. Most of the included 118 studies indicated that implementation efforts which involved educational efforts had an impact on knowledge, on attitudes, and to a lesser extent on practice change [30]. It is anticipated that our study will also result in improvements in awareness, use, and impact of our clinical practice guidelines due to our efforts to engage stakeholders in identifying barriers pre-implementation.

Challenges to date with our study have centered on obtaining ethics approval for our study at our central location as well as the eight sites across the country where focus groups will be completed. Due to staffing shortages within ethics departments, there have been delays in review and approval of our study. We have obtained ethics approval at our site and we are now going through the process of meeting ethical obligations at each of the other eight sites. We expect to have ethics approval at these other sites shortly and will begin recruitment.

In summary, more evidence is needed with respect to the impact of guideline implementation interventions and strategies. Guidelines are produced with the best of intentions; however, research indicates they have limited impact if active strategies are not used to enhance their uptake and integration into real-world practice. Our study will provide clinicians and other stakeholders with practical educational materials about the best available evidence on treating children and adolescents with EDs and will generate valuable evidence on the effectiveness of tailored implementation strategies to improve guideline integration efforts.

### Supplementary Information

**Additional file 1.** Focus group interview guide.

** Additional file 2.** Pre- and post-survey.

## Abbreviations

CPS: Canadian Pediatric Society
EDAC: Eating Disorders Association of Canada
EDs: Eating disorders
NIED: National Initiative for Eating Disorders
NEDIC: National Eating Disorders Information Centre
SQUIRE: Standards for QUality Improvement Reporting Excellence
TIDieR: Template for Intervention Description and Replication
