# Supplementary material for: A study protocol for implementing Canadian Practice Guidelines for Treating Children and Adolescents with Eating Disorders
Source: Implement Sci Commun. 2024 Jan 5;5:5. doi: 10.1186/s43058-023-00538-9 (PMC10768347; doi:10.1186/s43058-023-00538-9)
Supplement: Supplementary file 1 — Additional file 1. Focus group interview guide. [file 43058_2023_538_MOESM1_ESM.docx]

Supplemental File 1. Focus Group Guide

**Focus Group Qualitative Interview**— Implementing Canadian Practice Guidelines for Treating Children and Adolescents with Eating Disorders

The Canadian Practice Guidelines for treating Children and Adolescents with Eating Disorders were published in Feb 2020 in the Journal of Eating Disorders. These guidelines describe the evidence base for many treatments including psychotherapy and medications, as well as level of care. They provide recommendations for clinicians. A Virtual Care Addendum was published in April 2021. This focus group interview is meant to explore your views on these guidelines, as well as barriers and facilitators to adoption.

**Introductions**

Please describe the setting you work in OR your experience with eating disorders.

**General Questions**

Prior to today, were you aware of these guidelines?

Do you have any overall impressions of these guidelines?

In any way have they influenced your clinical practice or the care that you have received?

**Barriers and Facilitators**

From your perspective, what have been some of the barriers to adopting these guidelines in your environment?

*Prompt* – personal (attitude/beliefs), guideline (length), external factors (system issues, waiting lists)

What have been the facilitating factors that have helped the adoption of these guidelines in your setting?

*Prompt* – personal (individual/team buy-in), guideline (content, importance), external factors (system buy-in)

**Implementation Strategy**

What factors might aid in the implementation of these guidelines in your setting?

Who might be an appropriate messenger?

What would be the key messages?

Who would be an appropriate audience for these guidelines?

What infrastructure or technology might be needed to implement the guidelines?

How could we evaluate the uptake of these guidelines in your setting?

How can we ensure that our guidelines are adopted in remote and hard to serve areas that do not have dedicated eating disorder programs?

**EDI**

How can equity deserving groups (Black, Indigenous, racialized, and gender diverse groups) be reached by these guidelines?

Thank you for your participation!
