# Supplementary material for: A study protocol for implementing Canadian Practice Guidelines for Treating Children and Adolescents with Eating Disorders
Source: Implement Sci Commun. 2024 Jan 5;5:5. doi: 10.1186/s43058-023-00538-9 (PMC10768347; doi:10.1186/s43058-023-00538-9)
Supplement: Supplementary file 2 — Additional file 2. Pre- and post-survey. [file 43058_2023_538_MOESM2_ESM.docx]

Supplemental File 2. Pre and Post Implementation Survey

**Implementing Canadian Practice Guidelines for Treating Children and Adolescents with Eating Disorders**

Pre - Survey

Thank you for participating in our survey to study the implementation of Canadian Practice Guidelines for Treating Children and Adolescents with Eating Disorders. After completing this survey we will contact you in six months to complete the survey again.

*Canadian Practice Guidelines for treating Children and Adolescents with Eating Disorders were published in Feb 2020 in the Journal of Eating Disorders. These guidelines describe the evidence base for many treatments including psychotherapy and medications, as well as level of care. They provide recommendations for clinicians. A Virtual care addendum was published in April 2021.*

*This survey is meant to explore your awareness of these guidelines, as well as barriers and facilitators to their uptake.*

Demographics

Age

Sex M or F

Gender – Man, Woman, Trans Male, Trans Female, Nonbinary, Other

Ethnicity – White, Black, Indigenous, Hispanic, Asian, Other

Experience

Work in Specialized Eating Disorder Program

Work in Specialized Mental Health Program

Work in Community Mental Health Setting

Work in Primary Care

Lived experience with an eating disorder myself

Lived experience as a caregiver for a person with an eating disorder

If Work option selected:

Do you work specifically with children and/or adolescents with eating disorders Yes/No

Approximately how many patients with eating disorders did you see in the last year?

If lived experience option selected:

How long ago were you/your child diagnosed with an eating disorder?

General Questions

Prior to this survey were you aware of these guidelines?

1. No awareness
2. Little awareness
3. Moderate awareness
4. High awareness
5. Very high awareness

Prior to this survey, had you made use of these guidelines in your care or practice?

1. No use
2. Little use
3. Moderate use
4. High use
5. Very high use

Prior to this survey, had these guidelines had an impact on your care or practice?

1. No impact
2. Little impact
3. Moderate impact
4. High impact
5. Very high impact

Barriers and Facilitators

From your perspective, what are some of the barriers to adopting these guidelines in your environment?

|  | 1 – disagree strongly | 2- disagree | 3- neutral | 4- agree | 5 -agree strongly |
| --- | --- | --- | --- | --- | --- |
| Personal attitudes |  |  |  |  |  |
| No time to read them |  |  |  |  |  |
| Guideline is too long |  |  |  |  |  |
| Waiting lists are too long to implement the recommendations |  |  |  |  |  |
| Program does not support the recommendations |  |  |  |  |  |
| Lack of awareness |  |  |  |  |  |

Are there any other barriers you wish to comment on:

What are some facilitating factors that could help the adoption of these guidelines?

|  | 1 – disagree strongly | 2- disagree | 3- neutral | 4- agree | 5 -agree strongly |
| --- | --- | --- | --- | --- | --- |
| Personal attitudes |  |  |  |  |  |
| Time to read them |  |  |  |  |  |
| Guideline could be shortened |  |  |  |  |  |
| Waiting lists become shorter in order to implement the recommendations |  |  |  |  |  |
| Program would support the recommendations |  |  |  |  |  |
| Increase in awareness with education |  |  |  |  |  |

Implementation Strategies

What additional factors might aid in the implementation of these guidelines in your setting?

How can equity deserving groups (Black, Indigenous, racialized, and gender diverse groups) be reached by these guidelines?

Thank you for your participation!

Post - Survey

Thank you for participating in our survey to study the implementation of Canadian Practice Guidelines for Treating Children and Adolescents with Eating Disorders.

*Canadian Practice Guidelines for treating Children and Adolescents with Eating Disorders were published in Feb 2020 in the Journal of Eating Disorders. These guidelines describe the evidence base for many treatments including psychotherapy and medications, as well as level of care. They provide recommendations for clinicians. A Virtual care addendum was published in April 2021.*

*This survey is meant to explore your awareness on these guidelines, as well as barriers and facilitators to their uptake.*

Participant ID#. XXXX

General Questions

Within the last six months were you made aware of these guidelines?

1. No awareness
2. Little awareness
3. Moderate awareness
4. High awareness
5. Very high awareness

Within the last six months, have you made use of these guidelines in your care or practice?

1. No use
2. Little use
3. Moderate use
4. High use
5. Very high use

Within the last six months, have these guidelines had an impact on your care or practice?

1. No impact
2. Little impact
3. Moderate impact
4. High impact
5. Very high impact

Implementation Strategy

What aspects of our implementation strategy did you find effective?

|  | 1 – disagree strongly | 2- disagree | 3- neutral | 4- agree | 5 -agree strongly |
| --- | --- | --- | --- | --- | --- |
| Guideline Synopsis |  |  |  |  |  |
| Educational Video |  |  |  |  |  |
| Patient Guide |  |  |  |  |  |
| Increase in Team Buy-In about the guidelines |  |  |  |  |  |
| Increased awareness within your team about the guidelines |  |  |  |  |  |

Any other comments about the implementation strategy?

Do you feel equity deserving groups were reached by these guidelines? Are there other strategies that we could use?

Thank you for your participation!
